# Health financing in Africa: overview of a dialogue among high level policy makers

**DOI:** 10.1186/1753-6561-5-S5-S2

**Published:** 2011-06-13

**Authors:** Luis Gomes Sambo, Joses Muthuri Kirigia, Georges Ki-Zerbo

**Affiliations:** 1 World Health Organization, Regional Office for Africa, B.P. 06, Brazzaville, Congo

## Abstract

**Background:**

Even though Africa has the highest disease burden compared with other regions, it has the lowest per capita spending on health. In 2007, 27 (51%) out the 53 countries spent less than US$50 per person on health. Almost 30% of the total health expenditure came from governments, 50% from private sources (of which 71% was from out-of-pocket payments by households) and 20% from donors. The purpose of this article is to reflect on the proceedings of the African Union Side Event on Health Financing in the African continent.

**Methods:**

Methods employed in the session included presentations, panel discussion and open public discussion with ministers of health and finance from the African continent.

**Discussion:**

The current unsatisfactory state of health financing was attributed to lack of clear vision and plan for health financing; lack of national health accounts and other evidence to guide development and implementation of national health financing policies and strategies; low investments in sectors that address social determinants of health; predominance of out-of-pocket spending; underdeveloped prepaid health financing mechanisms; large informal sectors vis-à-vis small formal sectors; and unpredictability and non-alignment of majority of donor funds with national health priorities.

Countries need to develop and adopt a comprehensive national health policy and a costed strategic plan; a comprehensive evidence-based health financing strategy; allocate at least 15% of the national budget to health development; use GFATM and PEPFAR funds for health systems strengthening; strengthen intersectoral collaboration to address health determinants; advocate among donors to implement the Paris Declaration on Aid Effectiveness and its Accra Agenda for Action; ensure universal access to health services for pregnant women, lactating mothers and children aged under five years; strengthen financial management capacities; and develop prepaid health financing systems, especially health insurance to complement tax funding.

In addition, countries need to institutionalize national health accounts; undertake feasibility studies of various health financing mechanisms; and document and share best practices in health financing.

**Conclusion:**

There was consensus that every country ought to have an evidence-based comprehensive health financing strategy with a road map for attaining universal health service coverage vision; and increase physical and financial access by pregnant women, lactating mothers and by children under five years to quality health services.

## Background

The African Union Commission held an Official Side Event on Health Financing in Africa on 24 July 2010 as part of the 15th Ordinary Assembly of the African Union in Kampala, Uganda. The objectives of the Side Event were to (i) identify the reasons for the current poor state of health financing in Africa and define what could be done by both African and global stakeholders to improve the situation and (ii) make a case for health investment and financing, especially within a continuum of care for maternal, infant and child health.

The session mainly involved presentations, panel discussion and open public discussion with ministers of health and finance from the African continent. The panellists included the Minister of Health of Ethiopia, the Minister of Finance of Sierra Leone, the Regional Director of the WHO Regional Office for Africa, the acting Human Development Director of the World Bank, and the Director of Partnerships at the Global Fund to Fight AIDS, Tuberculosis and Malaria (GFATM). Two presentations were made by representatives of the Harmonization for Health in Africa (HHA) initiative (which is made up of the African Development Bank, Joint United Nations Programme on HIV/AIDS, United Nations Children's Fund, United Nations Population Fund, World Health Organization and World Bank) and the African Public Health Alliance (APHA). These presentations were aimed at stimulating discussion.

This article reflects on the proceedings of the African Union Side Event on Health Financing.

## Summary of presentations

### Presentation by Harmonization for Health in Africa

In its presentation, entitled “Investing in Health for Africa” [1], HHA first highlighted the bleak situation of health in Africa. Although Africa had only 12% of the world’s population in 2008, it bore about 52% of the world’s burden of maternal and child deaths, 89% of malaria deaths, 76% of HIV/AIDS deaths, 46% of deaths from childhood diseases, and 34% of perinatal deaths associated with prematurity and low birth weight, birth asphyxia, birth trauma and neonatal infections, and other conditions [2]. Of the total of 10.951 million deaths in Africa in 2008, 65% were from communicable diseases, maternal and perinatal conditions, and nutritional deficiencies; 28% were from non-communicable conditions; and 8% were from injuries [2]. Thus, Africa is faced with the double burden of communicable and non-communicable diseases.

Even though Africa has the highest disease burden compared with other regions, it has the lowest per capita spending on health, partly due to its low gross domestic product and partly due to absence of national health care financing strategies. In 2007, 27 (51%) out the 53 countries spent less than US$50 per person on health [3]. About half of the health expenditure in Africa is from private sources.

In a United Nations General Assembly Resolution [4,5] 40 years ago, developed countries committed to allocate at least 0.7% of their gross national product (GNP) to Official Development Assistance (ODA). Development aid has been increasing but falls far short of what is needed and what has been promised. In 2008 only 5 of the 24 members of the Development Assistance Committee (DAC) allocated at least 0.7% of gross national income to official development assistance [6]. In 2008 DAC countries allocated in total 0.31% of their gross national income to ODA, which is below the 0.54% needed by developing countries to achieve their Millennium Development Goals (MDGs) [7]. Also, most of donor support for health is unpredictable, tied, and not aligned and harmonized with the health priorities and systems of the African countries.

These concerns notwithstanding, external aid is an important source of health spending in Africa. In 2007, for example, external resources for health constituted 20.4% of total expenditure on health in the continent [3].

There is need for more domestic and external investments in health for Africa focussing on [8]:

• Supporting health systems to develop evidence-based policies (including in the areas of policy analysis, policy dialogue and evidence-based planning and budgeting);

• Governance and accountability;

• Service demand and use, particularly reducing financial barriers to use of services, improving people’s awareness of services and encouraging health seeking behaviour;

• Service provision, particularly providing a larger and better skilled workforce; making essential health commodities available and accessible; improving health information management systems; upgrading, equipping and maintaining the health infrastructure; and improving quality and availability of health services, including community services.

According to HHA estimates, investing an additional average of US$21 to US$36 per capita per year over five years (2011–2015) would save 3.1 million lives in Africa (of which 90% would be among mothers and children), prevent between 3.8 million and 5.1 million children from stunting, build an additional 58,268 to 77,100 health facilities, and produce an additional 2 million to 2.8 million health workers in Africa. In short, the economic gains in 2015 would be up to $100 billion from that additional health investment [8,9].

The HHA presentation argued that the fiscal space for additional health investments at the country level could be increased through increases in ODA or tax revenues (especially in those countries where revenue from taxes constitutes less than 15% of GDP), reprioritization of public resource allocation from low to high priority sectors, and increases in public sector efficiency [10]. More resources for health development can be realized through increased efficiency of private spending, for example by channelling current household out-of-pocket payments through national risk pooling health insurance mechanisms as in Ghana and Rwanda. Establishment of the Health Systems Funding Platform for the Global Fund, GAVI Alliance, the World Bank and others may enable the three biggest health funds to work together more efficiently to coordinate, mobilize, streamline and channel the flow of existing and new international resources to support national health strategies [11].

In concluding, the presenters stated that health MDGs were ambitious and even in a conservative scenario would be challenging to implement without injection of new substantive finances. Sub-Saharan Africa countries require increased and better allocated domestic and external funding for strengthening their national health systems in order to achieve the MDGs. Since most of the resources are to come from country contributions, there is need for domestic advocacy to raise attention among national budgeting processes and to channel private household spending through risk pooling and sharing mechanisms such as social health insurance and tax-funded services. External aid is catalytic and needs to focus on results and efficiency gains.

### Presentation by the African Public Health Alliance

APHA underscored the fact that investment in health was arguably the most crucial investment any government could make, since health was a fundamental human right, [12] and a long and healthy life was a prerequisite for human capital development, high economic productivity and sustainable economic development [13,14]. Cognisant of the high disease burden and the importance of investing in health, African Heads of State and Government in 2001 committed in Abuja to allocate at least 15% of their countries’ annual government budgets to the health sector [15]. By the end of 2007, only 3 countries (Liberia, Rwanda and Tanzania) had fulfilled the Abuja commitment and only 30 out of 53 countries were allocating at least US$34 per person per year to health [3] as recommended by the WHO Commission for Macroeconomics and Health [16].

In order to achieve the health MDGs, in addition to increasing investments in health, Africa needs also to increase investments into sectors that address the social determinants of health such as education, food, water, sanitation and shelter, which also are under funded. For example, in 2008 only 61% of Africa’s population used improved drinking water sources, only 34% used improved sanitation, and as high as 78% used solid fuels, contributing to environmental pollution and increases in prevalence of respiratory diseases [3].

The APHA presentation concluded with an appeal to ministers of finance and chairs of parliamentary finance and budget committees in Africa to fulfil the commitment by Heads of State and Government to allocate at least 15% of national budgets to the health sector and also allocate to health at least US$34 per person per year.

## Discussion

### Current state of health financing

Participants noted that Africa’s per capita spending on health was the lowest (amid escalating health services costs) worldwide, and that expenditure on maternal and child health were grossly inadequate. Majority of the countries have not met the African Heads of State and Government commitment to allocate at least 15% of annual national budgets to health sectors. For a sizeable number of countries, the total health spending is less than the bare minimum of US$34 per person per year recommended by the WHO Commission for Macroeconomics and Health. In 2007 almost 30% of the total health expenditure came from governments, 50% from private sources (of which 71% was from out-of-pocket payments by households) and 20% from donors [3]. The participants expressed concern that most donor funds were still unpredictable and not aligned with national health priorities or harmonized with national systems.

It was observed that besides the critical shortages of investments in health, the current situation was also characterized by inefficient use of existing resources, weak mechanisms for coordinating partner support for the implementation of national health policies and strategic plans, and lack of effective social protection mechanisms to ensure equitable access to health care.

### Reasons for the current state of health financing

The panellists and participants attributed the current state of health financing to lack of clear vision and plan for health financing; lack of national health accounts and other evidence to guide development and implementation of national health financing policies and strategies; low investments in other sectors that address social determinants of health such as water and sanitation, food security, housing, and road safety; predominance of un-pooled out-of-pocket spending; underdeveloped prepaid health financing mechanisms, especially health insurance; large informal sectors vis-à-vis small formal sectors, which limits amounts of tax revenues to governments; limited evidence of cost-effectiveness of investments in health; and sizeable amounts of donor funds focussed on disease programmes and specific interventions without sufficient investment in programme integration, intersectoral collaboration and health systems strengthening.

The overall result of these challenges is weak health systems leading to poor health indicators and slow progress towards achievement of the health MDGs.

### What can be done to improve the state of health financing in Africa?

Various panellists and participants acknowledged that since countries in the continent were at different stages of development and political stability, it may not be possible to have a set of solutions that were universally applicable. Thus, the applicability of actions emanating from the debate for improving the current health financing situation would depend on each country’s context. The actions can be grouped as follows.

#### Policy and management

A number of actions were identified relating to health policy and management:

• The countries need to develop and adopt a comprehensive national health policy (NHP) and a costed strategic plan that is integrated into the overall development strategy.

• Every country needs to have a comprehensive evidence-based health financing strategy that reflects revenue collection, revenue pooling and financial risk management, and strategic purchasing of health services.

• Strong advocacy among parliamentarians and ministers of finance at various forums is necessary to fulfil the Heads of State and Government pledge to allocate at least 15% of the national budget to health development.

• Countries ought to use GFATM and PEPFAR (US President’s Emergency Plan for AIDS Relief) funds to address health system issues.

• Strengthening of intersectoral collaboration for improving health determinants is a necessary prerequisite for health development.

• There is need to advocate among donors to stick to the spirit and content of the Paris Declaration on Aid Effectiveness [17] and its Accra Agenda for Action [18].

• In the spirit of the Paris Declaration, African countries need to demonstrate accountability in the use of both domestic and external funds in delivering concrete results.

• National governments should improve coordination of health and development partners at the country level to support national health policies and strategic plans.

• Partners should channel their support for health sectors through general budget support for ease of implementation, monitoring and evaluation, and efficiency.

• Partners should replenish the resources of the GFATM, and GFATM should open a window for maternal and child health.

• Countries should ensure universal access to health services for pregnant women, lactating mothers and children under five years.

• It is vital to sustain the current momentum in support for mechanisms such as the International Health Partnership Plus (IHP+) and HHA to promote harmonization and alignment with the national health policies and health sector strategic plans.

#### Capacity building and strengthening

Two actions were identified with regard to capacity building:

• It is necessary for countries to strengthen financial management capacities, including competencies in budgeting, planning, accounting, auditing, and monitoring and evaluation at all levels.

• There is need to develop prepaid health financing systems (social health insurance, community health insurance and tax-funded systems) to raise more funds for health and cushion households against catastrophic out-of-pocket expenditures on health services.

#### Evidence including research and information

Concerning evidence for decision-making, three actions were proposed:

• There is need for countries to institutionalize national health accounts within health management information systems for better tracking of health expenditures and to use that information for advocacy with ministers of finance to increase investments in health.

• Countries will need to undertake exploratory studies to ascertain whether various approaches, such as performance- or results-based financing can actually increase the efficiency of public and private health-care sectors.

• There is need to document and share experience on how some countries like Rwanda have used GFATM funds to support scaling-up of coverage of community-based health insurance.

## Conclusion

Consensus emerged on several key actions. First, it is important for every country to have a comprehensive health financing strategy with a road map for attaining universal health service coverage vision. Second, the national health financing strategy will need to be informed by national health accounts analysis and strongly anchored in costed national health sector strategic plan. Third, countries ought to make concerted efforts to fulfil the promise to their citizens of allocating on a sustained basis at least 15% of the annual national budget to health and to use the amounts allocated equitably and efficiently. It was felt that this would give the African Union moral authority to nudge developed countries to fulfil their own commitments to Africa. And fifth, there is need for increasing physical and financial access of pregnant women, lactating mothers and children under five years of age to quality health services.

## Competing interests

The authors declare that they have no competing interests.
